# Decorin inhibits proliferation and metastasis in human bladder cancer cells by upregulating P21

**DOI:** 10.1097/MD.0000000000029760

**Published:** 2022-06-30

**Authors:** Hongjie Chen, Ziyi Wang, Ninggang Yang, Jun Zhang, Zhong Liang

**Affiliations:** a Department of Urology, the First People’s Hospital of Lanzhou, Gansu, Lanzhou, China; b Clinical Department of Integrated Traditional Chinese and Western medicine, Gansu University of Chinese Medicine, Gansu, Lanzhou, China.

**Keywords:** apoptosis, bladder cancer, decorin, p21, metastasis

## Abstract

Migration of bladder cancer (BC) cells poses a substantial threat to human health. It is critical to elucidate the mechanism of BC invasion and progression for surgical treatment and the prognosis of patients. Decorin is of interest as an anticancer treatment that can play a vital role in regulating tumorigenesis.

The effect of decorin expression on survival in clinical patients was screened and analyzed using bladder urothelial carcinoma data from the Cancer Genome Atlas (TCGA) database. The differential expression of transforming growth factor-β1 (TGF-β1) in tumors was compared against that of normal samples to analyze the correlation between them. MTT, flow cytometry, and Wound/Transwell assays were used to detect cell proliferation, cycle arrest, apoptosis, migration, and invasion.

Analysis of TCGA data showed that decorin expression was significantly lower in bladder urothelial carcinoma samples than in normal tissues, while TGF-β1 expression did not change significantly. We found that decorin was correlated with TGF-β1 expression in bladder urothelial cancer. In addition, decorin blocked the G1/S phase by upregulating p21 protein and inhibiting the expression of TGF-β1 and MMP2, promoting the occurrence of apoptosis and inhibiting the proliferation of human BC T24 cells. Moreover, decorin increased the adhesion of tumor cells in vitro, and effectively inhibited cell metastasis.

Decorin regulated the expression of TGF-β1 and MMP2 through p21 protein, promoted apoptosis and adhesion, and inhibited the proliferation and metastasis of BC cells.

## 1. Introduction

Human bladder cancer (BC) is a heterogeneous disease. It is one of the most common types of cancer worldwide, with approximately 550,000 newly-diagnosed cases every year.^[1]^ According to recently reported data, new cases of urinary BC form approximately 7%, and BC-related deaths constitute nearly 4% of all reported cases of cancer.^[2]^ Currently, the main treatment for BC is surgery. However, due to the high rates of recurrence and metastasis, the overall survival rate of patients with BC remains unsatisfactory.^[3]^ BC has a high recurrence rate, and approximately 25% of all patients with nonmuscle invasive bladder cancer (NMIBC) develop muscle-invasive BC following treatment or will eventually develop metastasis.^[4]^ In addition, BC is at risk of recurrence or more likely to lead to recurrence. Over the last few decades, novel therapeutic strategies have been developed, but the prognoses and survival rates of patients with cancer remain far from satisfactory. Therefore, the identification and development of new anticancer drugs can significantly improve treatment efficacy.^[5]^ Nevertheless, very little is known about the effects of these drugs on BC.

Decorin is a small molecule proteoglycan. It is a secreted multifunctional proteoglycan involved in cell adhesion, migration, and proliferation and participates in the regulation of cell growth and tissue remodeling processes.^[6]^Additionally, decorin plays an important role in suppressing the growth of various tumor cell lines. Previous studies have suggested that decorin may be a component of a feedback system that regulates cell growth.^[7]^ Moreover, decorin binds to transforming growth factor β (TGF-β), which in turn inhibits endothelial–mesenchymal transition and fibrosis.^[8]^ Studies have suggested that decorin is a multifunctional cytokine that regulates cell proliferation, differentiation, apoptosis, and migration.^[9]^ More importantly, it has been shown that decorin binds to TGF-β to inhibit the activity of TGF-β and increase the expression of p21 protein.^[10]^ In tumor cell-mediated angiogenesis, the expression of p21 was regulated largely at the transcriptional level by both p53-dependent and -independent mechanisms.^[11]^ MMP2 and MMP9 are closely associated with cancer metastasis because of their strong proteolytic activity in the extracellular matrix (ECM).^[12]^ It is widely believed that the effect of MMP2 on the ECM is closely associated with tumor invasion and metastasis. It has been reported that high expression of MMP2 promotes BC cell metastasis.^[13]^ TGF-β1 activates cell migration and invasion through MMP2 activity.^[14]^ Notably, the role of p21 in tumor cells has received increasing attention. Therefore, the objective of this study was to investigate the effect of decorin on the proliferation and metastasis of human BC cells (T24), and to further explore the potential regulatory mechanism affecting p21 expression.

## 2. Materials and Methods

### 2.1. Cell-culture and co-expression analysis

BC cells and human T24 cells (Cat No. 8200D301004) were purchased from American Type Culture Collection (ATCC; Manassas, VA). The cells were cultured with 5% CO_2_ at 37 °C in RPMI-1640 medium (Gibco, Carlsbad, CA) containing 10% fetal bovine serum (FBS; Gibco, Carlsbad, CA), 100U/mL penicillin, and 100 μg/mL streptomycin (Solarbio, Beijing, China). Human decorin/DECORIN protein (CatNo. 10189-HNAH; Sino Biological Inc., Beijing, China) was diluted with 1× PBS to a final concentration of 1.48 mg/mL and was kept frozen at −80 °C for further study. Geneexpression data for BC tissues were downloaded from The Cancer Genome Atlas (TCGA) database (https://tcga-data.nci.nih.gov/tcga/). Data of samples from 408 patients with bladder urothelial carcinoma (male, n = 297; female, n = 105; aged 61–85 years) and that of 19 normal (control) samples (male, n = 13; female, n = 6; aged 41–60 years) were obtained from the TCGA database. Decorin expression was analyzed using the R language package, and the analysis of decorin and TGF-β1 was obtained from the TCGA.

### 2.2. MTT assay

Cell viability was assessed using MTT assay. After completion of the cell-culture and co-expression analysis, followed by a gentle phenol red-free RPMI-1640 media wash, MTT at a final concentration of 0.5 mg/mL was added to the cells and the set-up was incubated at 37 °C for 2 hours. Absorbance was measured at 570 nm using a microplate reader (Bio-Rad, Hercules, CA). The data are presented as the percentage of surviving transfected cells vis-a-vis control cells (viability of control cells was considered to be 100%).

### 2.3. Cell cycle and apoptosis analysis

Cells were seeded into 6-well cell-culture plates for 24 hours, and decorin stimulation was given after every 24 hours of culture. Culture was discontinued at 72 hours from initiation. Cells were labeled with propidium iodide, and the cell cycle was determined by flow cytometry analysis (BD Biosciences, San Jose, CA). For the apoptosis assay, cells undergoing apoptosis and necrosis were measured using a PE Annexin V Apoptosis Detection Kit (BD Biosciences, Franklin Lakes, NJ). All flow cytometry data were analyzed using FACScan (BD Biosciences, Franklin Lakes, NJ).

### 2.4. Wound-healing, migration, and transwell invasion assay

Migration was assessed using a wound-healing assay. Cells were plated in 12-well dishes (5 × 10^4^ cells/well), and incubated in RPMI-1640 medium without FBS at 37 °C, reaching a confluence of 80%. Next, the cells were scratched across the surface of the well using a 10-µl pipette. After incubation at 37 °C for 24 hours, scratching was observed.

The migration and invasion of cells were examined using 24-well chambers with 8 μM pore size membrane, with (invasion) and without Matrigel (migration), as previously described.^[15]^ Briefly, 4 × 10^4^ cells were plated on the inserts and cultured at 37 °C in the upper chambers without serum. For the transwell assay, polycarbonate membranes with 8-μm pores (Corning, NY) were placed on 24-well Transwell plates (Corning, NY). Briefly, polycarbonate filters were coated with Matrigel at a concentration of 1 μg/ml and placed in a modified Boyden chamber. After trypsin-digestion, the cells (3 × 10^4^) were resuspended in RPMI-1640 medium containing 1% (v/v) FBS and were added to the top chamber. Culture medium containing 5% (v/v) FBS was then added to the bottom chamber. The invading cells in the lower chamber were treated with methanol and 0.1% crystal violet at room temperature. A light microscope (X7, Nikon; Tokyo, Japan) was used to photograph the membranes. Five fields (magnification ×100) of each sample were randomly photographed from each sample. Cell motility and migration were measured as the number of cells migrating from a defined area of the uncoated microfilter through micropores at 48 hours.

### 2.5. RNA extraction and RT-PCR assay

For mRNA expression, total RNA was extracted using TRIzol reagent (Invitrogen, Carlsbad, CA) according to the standard protocol. RNA concentration and quality were analyzed using a spectrophotometer (Nanodrop-2000, Thermo Fisher Scientific; Waltham, MA). RNA (1 μg) was used as a template for the reverse transcription reaction. First-strand cDNA was synthesized using the GoScript™ Reverse Transcription System (Promega, CA) according to the manufacturer instructions and then stored at −20 °C.

Following are the forward and reverse primer sequences, respectively:

p21-F: GTCACCGAGACACCACTGGAGGGT

p21-R: CTGAGCGAGGCACAAGGGTACAAG

The primers were synthesized by TsingKe Biological Technology (Xi’an, Shanxi, China). Quantitative real-time PCR (qRT-PCR) was performed using a SYBR Green Premix Ex Taq kit (TsingKe, Beijing, China) on an Mx3005p system (Agilent Technologies, Santa Clara, CA) following the manufacturer instructions. The data were analyzed using the 2^-ΔΔCT^ method, and β-actin was used as an internal control.

### 2.6. Western blot analysis

Cells treated with decorin were harvested and suspended in radioimmunoprecipitation assay (RIPA) lysis buffer (Solarbio, Beijing, China). Protein concentrations were determined using a bicinchoninic acid (BCA) protein assay kit (Solarbio, Beijing, China). The proteins were resolved on a 12% sodium dodecyl sulfate polyacrylamide gel (SDS-PAGE) and transferred to a polyvinylidene difluoride (PVDF) membrane (Millipore, USA). This was followed by blocking with PBS, containing 5% bovine serum albumin, and the PVDF membranes were incubated with p21 polyclonal antibody (Cat No. ab109199, 1: 5000), β-actin (Cat No. ab227387, 1: 5000), TGF-β polyclonal antibody (Cat No. 21898-1-AP, 1: 1000) and MMP2 (Cat No. 10373-2-AP, 1: 1000) overnight at 4 °C. Membranes were washed thrice with TBST and incubated with goat antirabbit IgG H&L (HRP) (Cat No. SA0001-2, 1: 5000). After washing with TBST 5 times, proteins were visualized using an enhanced chemiluminescence (ECL) western blot analysis system.

### 2.7. Enzyme-linked immunosorbent assay

For logarithmic growth, the cells were collected and plated into 6-well culture plates at a density of 2 × 10^5^ cells/well. Cell-culture supernatants in serum-free medium were homogenized and harvested 72 hours later and centrifuged at 1000 ×g for 10 minutes, and the level of TGF-β1 was subsequently determined using a commercially available ELISA kit (Cat No. EHC107b, NeoBioscience, China), according to the manufacturer instructions.

### 2.8. RNA interference assay

T24 cells were seeded in 6-well plates at 50%–70% confluence. The siRNAs were synthesized as follows: human p21: AGACCATGTGGACCTGTCA. These siRNAs (40–80 pmol/well) were transfected into T24 cells using Lipofectamine RNAiMAX according to the manufacturer instructions (Invitrogen, Thermo Fischer Scientific; Waltham, MA). All siRNAs used in this study were purchased from RiboBio Co., Ltd. (Guangzhou, China). The treated cells were incubated for 36 hours with siRNA and subjected to decorin treatment for 48 hours. Then, the cells were harvested and analyzed by western blotting.

### 2.9. Statistical analysis

GraphPad5.0 and SPSS 25.0 were used for statistically analyzing the data. The data shown are the mean ± SD. All experiments were repeated at least 3 times independently. *P* value, that represents significant difference, was evaluated by 2-tailed *t*-test (*P < 0.05, **P < 0.01, ***P < 0.001).

## 3. Results

### 3.1. Effect of decorin on survival of clinical patients and in vitro proliferation in T24 cells

**Figure 1.**
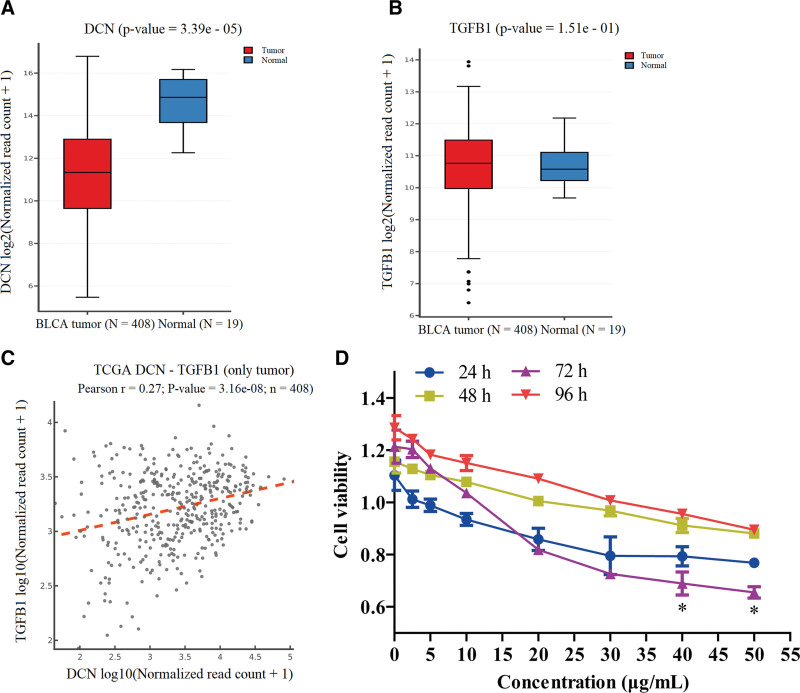
TCGA data analysis and in vitro proliferation. (A) Expression of decorin molecules in bladder urothelial carcinoma cells. (B) TGF-β1 expression in bladder urothelial carcinoma cells. (C) Correlation of decorin and TGF-β1 expressions in bladder urothelial carcinoma cells. (D) Effect of different concentrations of decorin on T24 cell proliferation.

The expression levels of decorin in 408 people with bladder urothelial carcinoma were analyzed using TCGA data. We found that decorin expression was decreased in tumor tissues compared to that in normal renal tissue (Fig. 1A), while the expression of TGF-β1 did not change significantly (Fig. 1B). These results showed a correlation between decorin and TGF-β1 expression in clinical samples (Fig. 1C).

To evaluate the interaction between decorin and T24 cells, cells were treated for 24 hours with decorin at concentrations of 0, 2.5, 5, 10, 20, 30, 40, and 50 μg/mL. Figure 1D showed that cell viability was reduced at different time points, which further indicated that decorin inhibited cell proliferation in a dose-dependent manner. Besides, these results showed the most significant inhibition of cell proliferation, and the cell viability was at the lowest level. When compared at 24 hours, no significant differences were observed between effects at 0 and 30 μg/mL decorin concentrations. However, 40 μg/mL and 50 μg/mL decorinsignificantly decreased cell viability (*P* < 0.05) and it reached the lowest level at 72 hours (Fig. 1D). The 50% inhibitory concentration was calculated to be 40.35 μg/mL. These data demonstrate that decorin inhibits cell proliferation.

### 3.2. Decorin changes cell cycle of T24 cells

**Figure 2.**
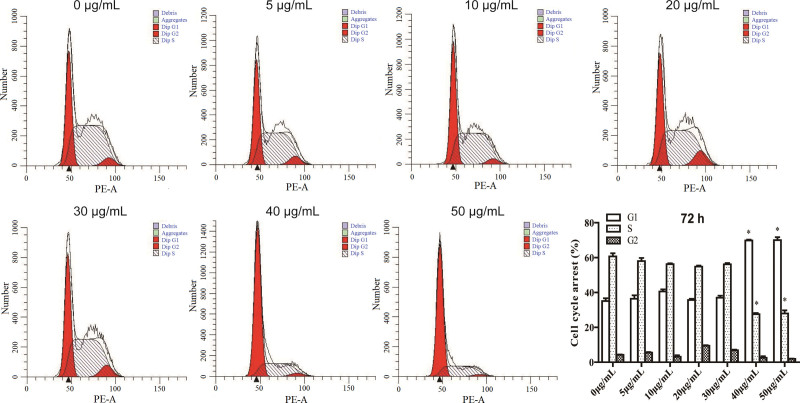
Cell cycles of T24 after treatment with decorin. The cells were seeded on 6-well plates for 24 hours, and stimulated with docorin (0 to 50 μg/mL) for 72 hours. Then the cell cycle was detected.

The results of cell cycle analysis showed that decorin arrested the T24 cell cycle in G1 phase, cell cycle G1 was significantly increased, but cell cycle S was decreased at 50 μg/mL compared with the control at 24 hours (*P* < 0.05) (Figure S1A, Supplemental Digital Content, http://links.lww.com/MD/G806). Decorin at concentrations 40 μg/mL and 50 μg/mL significantly boosted the G1 phase and weakened the S phase at 72 hours (*P* < 0.05) (Fig. 2). Decorin at 40 μg/mL and 50 μg/mL showed similar effects at 48 hours as well (Figure S1B, Supplemental Digital Content, http://links.lww.com/MD/G806). The number of G1 phase arrests was the highest, while that of the S phase was the lowest at 96 hours after decorin treatment (Figure S1C, Supplemental Digital Content, http://links.lww.com/MD/G806). Taken together, these data suggest that only the S and G1 phases were significantly changed, and the G2 phase remained unchanged throughout.

### 3.3. Decorin induces apoptosis in T24 cells

**Figure 3.**
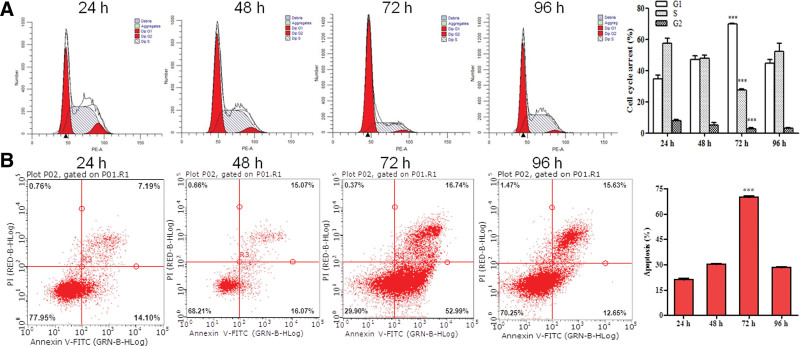
Apoptosis of T24 treated with decorin, as detected by flow cytometry. The cells were seeded on 6-well plates for 24 hours, and stimulated with docorin (0–50 μg/mL) for 72 hours. then the apoptosis was detected.

**Figure 4.**
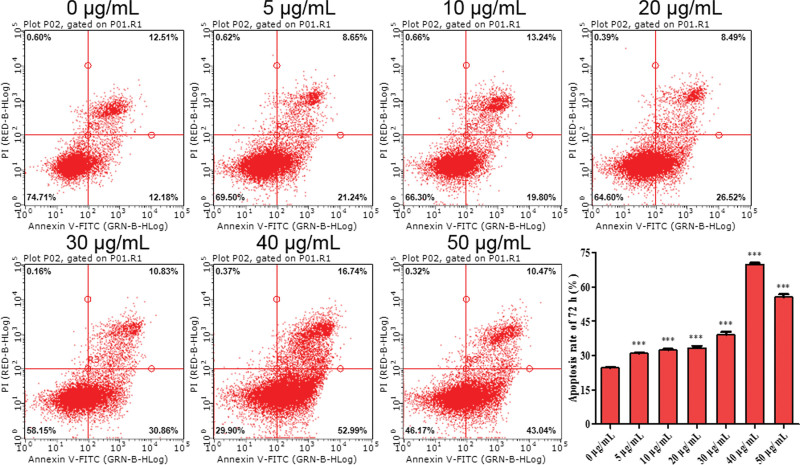
Cell cycle and apoptosis of T24 treated with decorin. (A) Cell cycle of different times treated with 40 μg/mL decorin. (B) Apoptosis of different times treated with 40 μg/mL decorin.

Flow cytometry was used to detect decorin-induced apoptosis. The analysis showed that the apoptosis rate of T24 clearly increased with decorin concentrations from 10 μg/mL to 50 μg/mL at 24 hours (*P* < 0.05) (Figure S2A, Supplemental Digital Content, http://links.lww.com/MD/G806). However, 30 μg/mL decorin also significantly increased apoptosis at 48 hours (*P* < 0.001), (Figure S2B, Supplemental Digital Content, http://links.lww.com/MD/G806). In addition, cell apoptosis was remarkably observed at 72 hours at decorin concentrations from 5 to 50 μg/mL, and it reached its maximum level at 40 μg/mL (Fig. 3). At 96 hours, the apoptosis rate was approximately consistent with that at 24 hours. However, 10 μg/mL decorin also significantly promoted apoptosis (Figure S2C http://links.lww.com/MD/G806). These results, in agreement with the MTT assay, further demonstrated that 40 μg/mL decorin also contributes to cell apoptosis, and duration of 72 hours was considered the most effective (Fig. 4).

### 3.4. Decorin downregulates TGF-β1 and upregulates p21 protein

**Figure 5.**
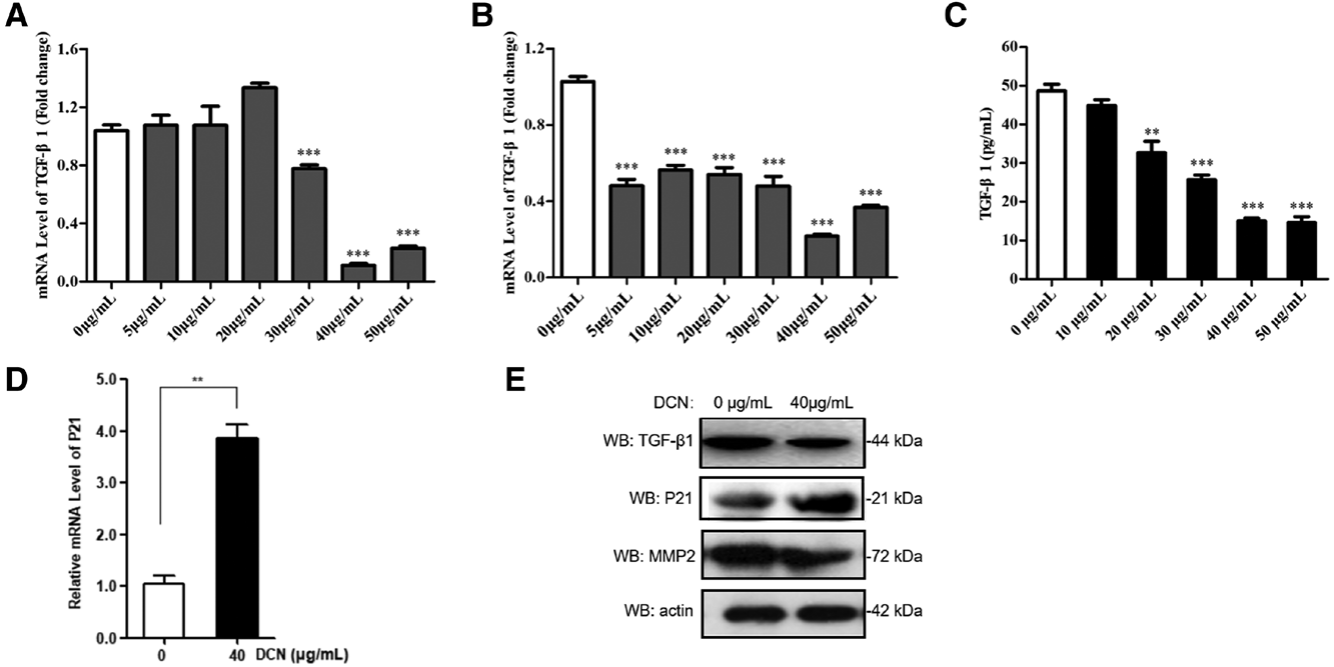
Decorin downregulated TGF-β1 and upregulated P21 protein. (A) TGF-β1 level assessed by qRT-PCR at 48 hours. (B) TGF-β1 level assessed by qRT-PCR at 72 hours. (C) TGF-β1 level determined by ELISA at 72 hours. (D) The expression of P21 detected by qRT-PCR at 48 hours. (E) Western blot analysis of TGF-β1, P21, and MMP2.

To further explore the potential regulatory mechanisms, we detected TGF-β1, MMP2, and p21. We found that decorin concentrations of 30, 40, and 50 μg/mL significantly reduced the expression of TGF-β1 at 48 hours, with the most obvious inhibition being at 40 μg/mL (Fig. 5A). When T24 cells were treated with decorin for 72 hours, the mRNA level of TGF-β1 was significantly inhibited by decorin (*P* < .001) (Fig. 5B). Additionally, ELISA analysis indicated that the amount of TGF-β1 in the supernatant at 72 hours was significantly reduced, approximately one-third lower than that after treatment with 40 μg/mL decorin, and that TGF-β1 expression decreased in a concentration-dependent manner; the most obvious inhibition being at 40 μg/mL (Fig. 5C). Interestingly, when compared to the control group (0 μg/mL), western blot analysis also showed that 40 μg/mL decorin actually decreased the expression of TGF-β1 (Fig. 5E). These data revealed that decorin downregulated TGF-β1 at both the transcriptional and translational levels. The protein levels of MMP2 were further inhibited by decorin (Fig. 5E).

To gain further insight into the mechanism by which proteins induce apoptosis, western blotting was performed. Compared to the control, the expression of p21 was induced by decorin at the transcriptional and translational levels (Fig. 5D–E), indicating that decorin may promote the expression of p21 to induce apoptosis.

### 3.5. Effect of decorin on adhesion, invasion, and migration of T24 cells

**Figure 6.**
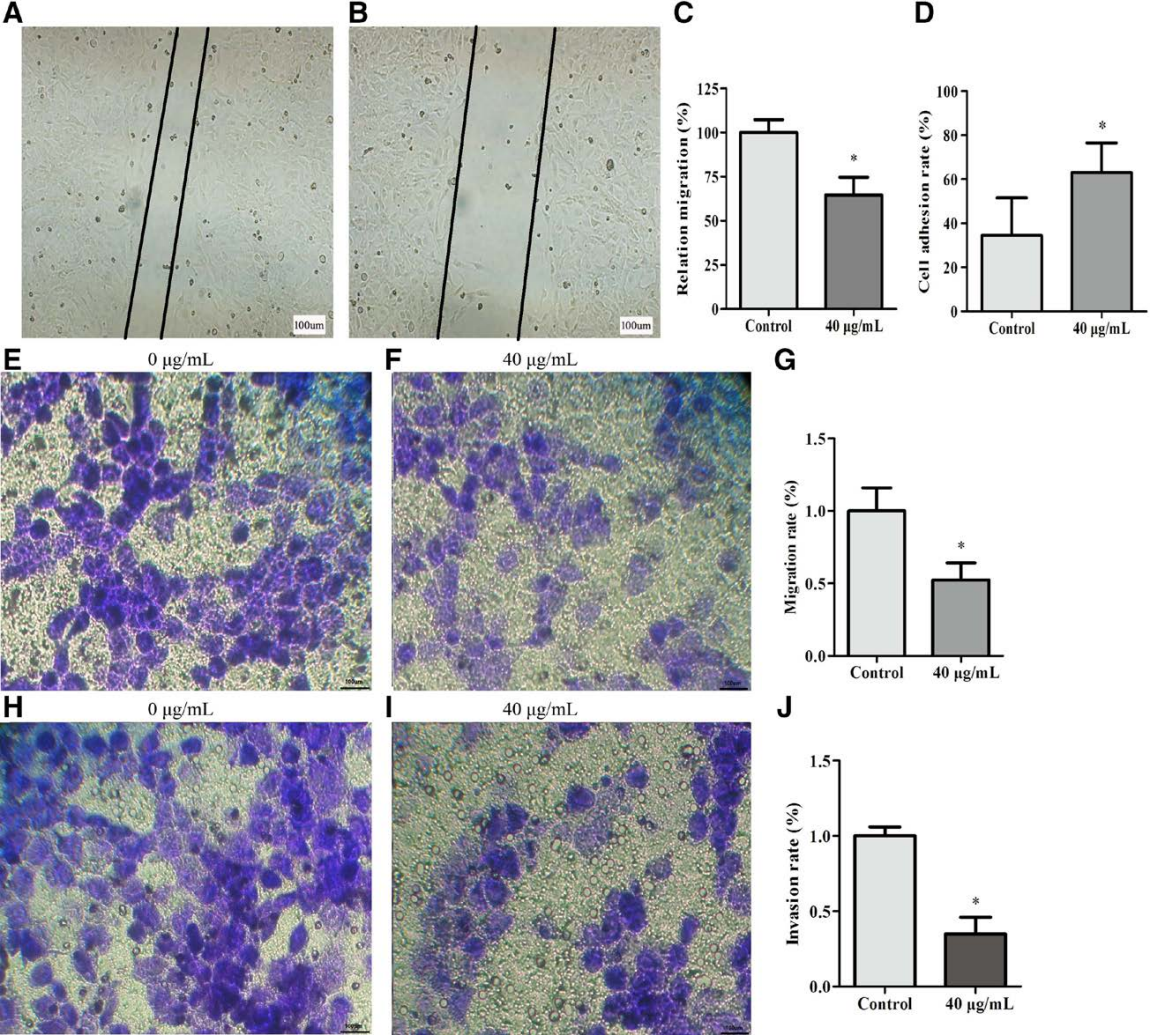
Effect of decorin on adhesion, invasion, and migration of T24 cells. (A) Migration of T24 treated with 0 μg/mL decorin. (B) Relative migration of T24 treated with 40 μg/mL decorin. (C) Relative migration rate. (D) Cell adhesion rate. (E) Migration of T24 cells treated with 0 μg/mL decorin. (F) Migration of T24 cells treated with 40 μg/mL decorin. (G) Migration rate. (H) Invasion of T24 cells treated with 0 μg/mL decorin. (I) Invasion of T24 treated with 40 μg/mL decorin. (J) Invasion rate.

To clarify the mechanism underlying inhibition of T24, we examined cell adhesion, invasion, and migration. Scratch assay revealed a significant decrease in cell migration at 72 hours after treatment with 40 μg/mL decorin (*P* < 0.05) (Fig. 6A–C). Adhesion results showed that T24 cells stimulated with 40 μg/mL decorin had a significant increase in cell adhesion at 72 hours compared to the control group (*P* < 0.05) (Fig. 6D). The transwell assay also revealed that 40 μg/mL decorin was able to reduce migration (*P*<0.05) (Fig. 6E–G). The invasion of T24 cells was markedly reduced after treatment with decorin (40 μg/mL) (Fig. 6H–J).

**Figure 7.**
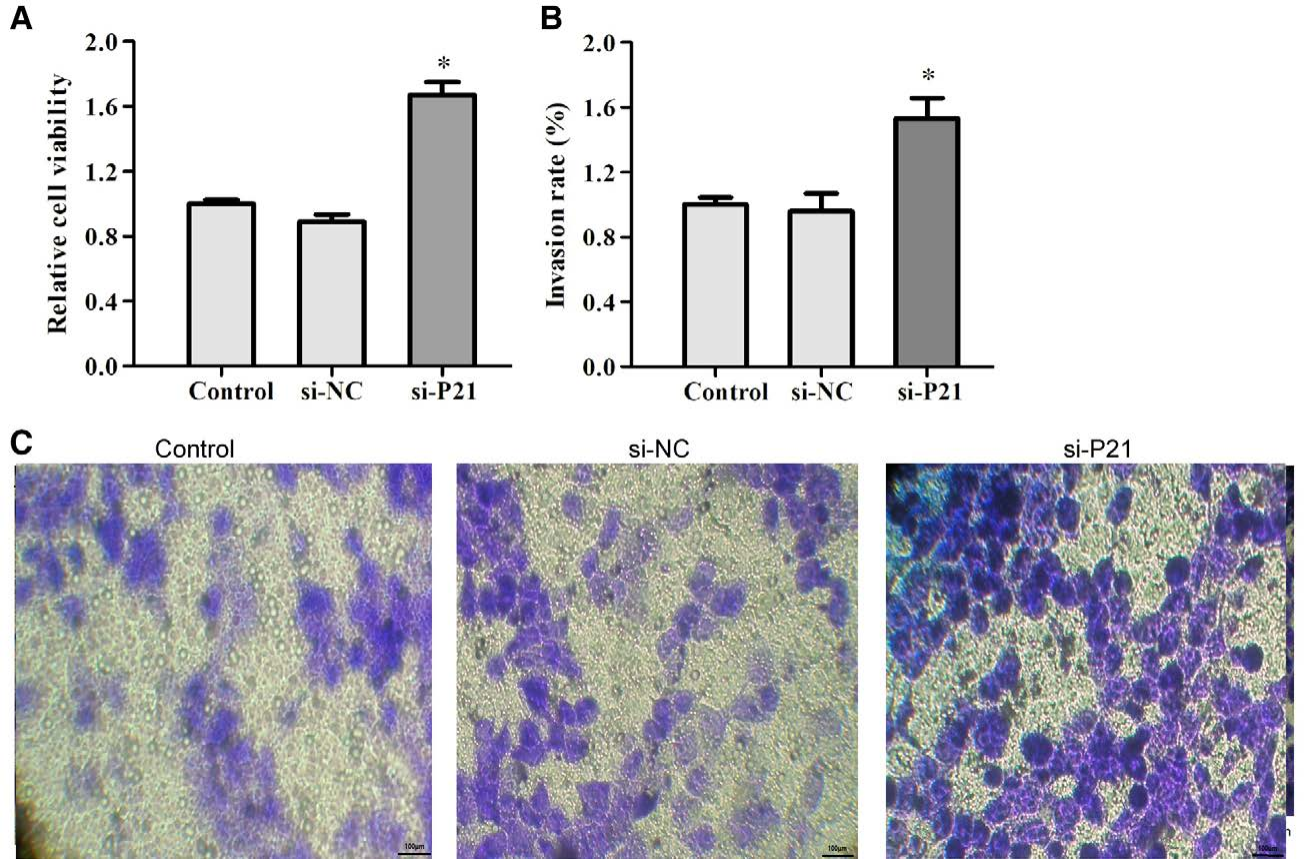
Knockdown of P21 induced cell proliferation and invasion rate. (A) Relative cell viability induced by si-P21. (B) Invasion rate induced by si-P21. (C) Invasion induced by si-P21.

To further clarify how decorin triggered proliferation and metastasis in T24 cells, we knocked down P21 expression. T24 cells were transfected with negative siRNA (NC) and 3 candidate P21 siRNAs for 48 hours. Knockdown efficiency was detected by western blotting. Western blot analysis showed that P21 siRNA2# efficiently downregulates P21 expression at the protein level. Thus, siRNA2# was selected for subsequent experiments (Figure S3, Supplemental Digital Content, http://links.lww.com/MD/G806). Stable knockdown of P21 induced cell proliferation and invasion rate (Fig. 7). Our study identified that P21 is responsible for cell apoptosis. Taken together, these results indicate that decorin promotes cell adhesion, suppresses the migration and invasion rate of T24 cells, and provides a novel mechanism of decorin in BC cells.

## 4. Discussion

BC is one of the most common malignant tumors of the urinary system and its incidence worldwide is very high.^[16]^ We examined cell proliferation, apoptosis, invasion, and migration induced by decorin in BC cells in vitro. We calculated the 50% inhibitory concentration of decorin on BC cells to be 40.35 μg/mL, and most notable results were observed at 72 hours of decorin treatment. This suggests that treatment with 40 μg/mL decorin for 72 hours is optimal for further study of BC cells.

One hypothesis is that adenovirus-mediated gene transfer leads to very high production of decorin, *and* that decorin pro-apoptotic effects require high decorin concentrations.^[17]^These results are consistent with those of this study. Decorin, a member of the SLRP family, decreases malignant transformation of tumor cells.^[18]^ Researchers have identified that while genetic ablation of decorin permits tumor growth, forced expression of decorin inhibits tumor growth.^[19]^

Additionally, our data showed that decorin inhibited the cell cycle and cell survival processes. The protein p21 is a direct target of the p53 protein, and p21 is responsible for p53-mediated G1 cell-cycle arrest. Decorin in tumor suppression is also associated with epidermal growth factor receptor (EGFR) and the cell cycle inhibitors p21 and p27.^[6]^Overexpression of decorin A significantly elevated the expression of apoptosis-related genes and decorin overexpression elevated proliferation-related genes. Decorin B played important roles in the promotion of pancreatic cancer (PC).^[20]^ Our study suggests that decorin induces apoptosis in T24 cells. It may be beneficial for the induction of tumor suppressors and BC. Increased decorin expression has also been shown to attenuate the migration of colorectal cancer cells and promote apoptosis.

The tumor microenvironment contains noncellular components, especially the ECM, which is composed of a variety of proteins, proteoglycans, and polysaccharides.^[21,22]^ ECM is the first vital barrier in tumor invasion and metastasis. The main component of ECM is type IV collagen. This, together with the important role of MMP2 in the degradation of type IV collagen, suggests MMP2 as a key enzyme involved in invasion and metastasis of cancer.^[23]^ Research also suggests increased accumulation of decorin within the ECM, complexed with latent TGF-β1^[24]^ Decorin has previously been shown to inhibit tumor angiogenesis and tube formation, although other studies have proposed an important role for decorin in wound healing.^[25]^ In several studies, decorin has been found to have a tumor suppressor role,^[26,27]^ while others have correlated decorin with increased tumor invasiveness, metastasis, and angiogenesis.^[28,29]^ Many studies have shown that decorin is a typical SRLP that can naturally maintain collagen fiber assembly. In this study, we found that a high concentration of decorin can significantly increase the adhesion between BC cells, as well as reduce the migration and invasion of cells. This may indicate a role of decorin in mediating the regulation of the expression of adhesion metastasis-associated molecules (E-cadherin and MMPs) and inhibiting tumor metastasis. Researchers have demonstrated that decorin suppresses tumorigenesis, invasion, and metastasis in inflammatory breast cancer.^[30]^ It may act as a tumor suppressor gene during tumor development and progression.^[31]^ It is a highly specific in vivo inhibitor of TGF-β as decorin and biglycan bind to and inhibit TGF-β activity.^[32]^ It sequesters and reduces the stromal bioavailability of TGF-β, which is therefore incapable of initiating downstream signaling.^[33]^ Decorin was found to inhibit TGF-β1 expression in BC cells and increase adhesion between tumor cells.^[34]^ This may demonstrate that it blocked the signaling pathway downstream of TGF-β1 and regulated the expression of adhesion-related molecules (E-cadherin, MMPs, etc), thereby promoting the adhesion between tumors and preventing tumor metastasis to distant sites. MMP2 degrades type IV collagen and promotes epithelial–mesenchymal transition and metastasis.^[35]^ This is in concordance with the results obtained in the present study, which indicated that decorin may inhibit the invasion and metastasis of BC by reducing MMP2 expression.

Many studies have shown that decorin can inhibit tumor metastasis and invasion.^[36,37]^ Here, we report that decorin expression can directly reverse the action of TGF-β1 on the immune system and induce the proliferation of immunocompetent cells possessing potent antitumor activity. Decorinarrests the cell cycle by downregulating the expression of p21.^[38]^ Previous research has shown that decorin overexpression could dramatically inhibit renal cell carcinoma (RCC) cell line proliferation, migration, and invasion by upregulating p21 and E-cadherin.^[39]^ Tumorigenesis is associated with the inactivation of p21, a protein downstream of p53 that regulates metastasis. Our study also showed that decorin decreased the expression of TGF-β1 and MMP2 and promoted the expression of p21. It is of interest that reduced p21 protein levels were accompanied by increased p21 mRNA levels, suggesting a translational down-regulation of p21 during cystogenesis. In this study, it was confirmed that decorin could increase G1/S phase arrest in T24 cells, change the cycle progression of BC cells, and promote the occurrence of apoptosis. Our study also illustrates that in BC, decorin promotes apoptosis and inhibits proliferation. Western blot analysis also confirmed that down-regulation of P21 was increased by decorin treatment at the protein level. Decreased decorin levels promote metastasis of colon cancer cells.^[40]^ High decorin expression can inhibit the invasion and metastasis of BC.^[41]^ These results were confirmed in our conclusions. Therefore, decorin is considered a tumor suppressor that affects cell proliferation, apoptosis, adhesion, invasion, and migration in some cancer cells. Understanding the mechanism of BC in T24 cells has shown potential for the development of cancer drugs for prevention and treatment.

In conclusion, we found that decorin enhanced cell apoptosis, endothelial cell attachment, migration, and differentiation of T24 cells. Decorin further downregulated the expression of TGF-β1 and MMP2 and inhibited its process. Knockdown of P21 induces cell proliferation and invasion. These results provide a novel insight into the mechanism of decorin in T24 cells and indicate that decorin could adversely affect the viability and apoptosis of T24 cells in vitro by upregulating the production of p21. These findings will help inform further research into BC and hopefully to help improve clinical prognosis in the future.

## Authors’ contributions

HJ C participated in the conception, design, and drafting of the manuscript. ZY W and NG Y conducted the data analysis. J Z and Z L contributed to editing the English text of this manuscript. All authors have read and approved the final submitted manuscript.

## Acknowledgments

We thank the Innovation and Entrepreneurship Project of Lanzhou and our colleagues in the First People’s Hospital of Lanzhou for their helpful comments.

## Supplementary Material


