# Supplementary figures and images for: Decorin inhibits proliferation and metastasis in human bladder cancer cells by upregulating P21
Source: Medicine (Baltimore). 2022 Jun 30;101(26):e29760. doi: 10.1097/MD.0000000000029760 (PMC9239591; doi:10.1097/MD.0000000000029760)

Figure S1

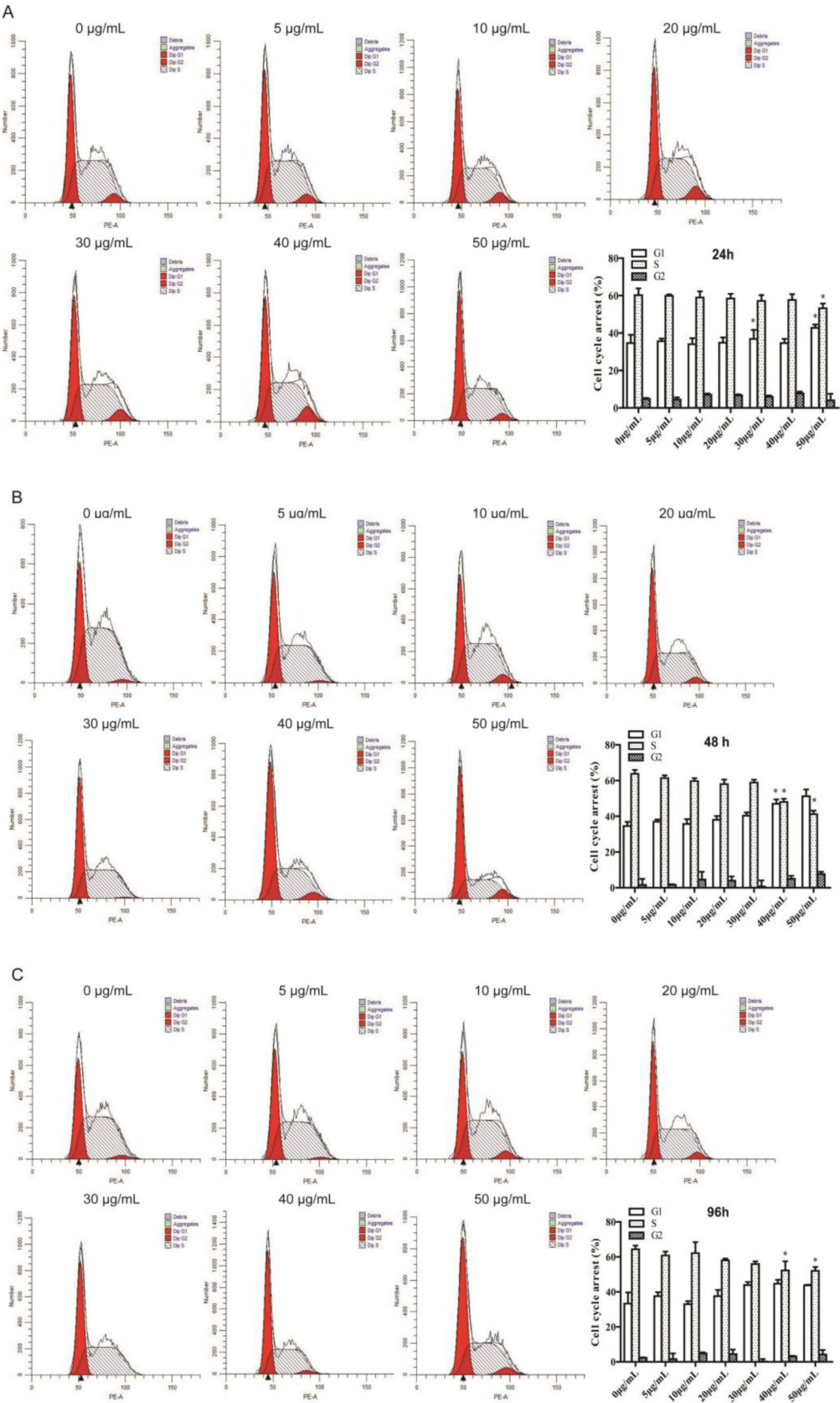

Figure S1

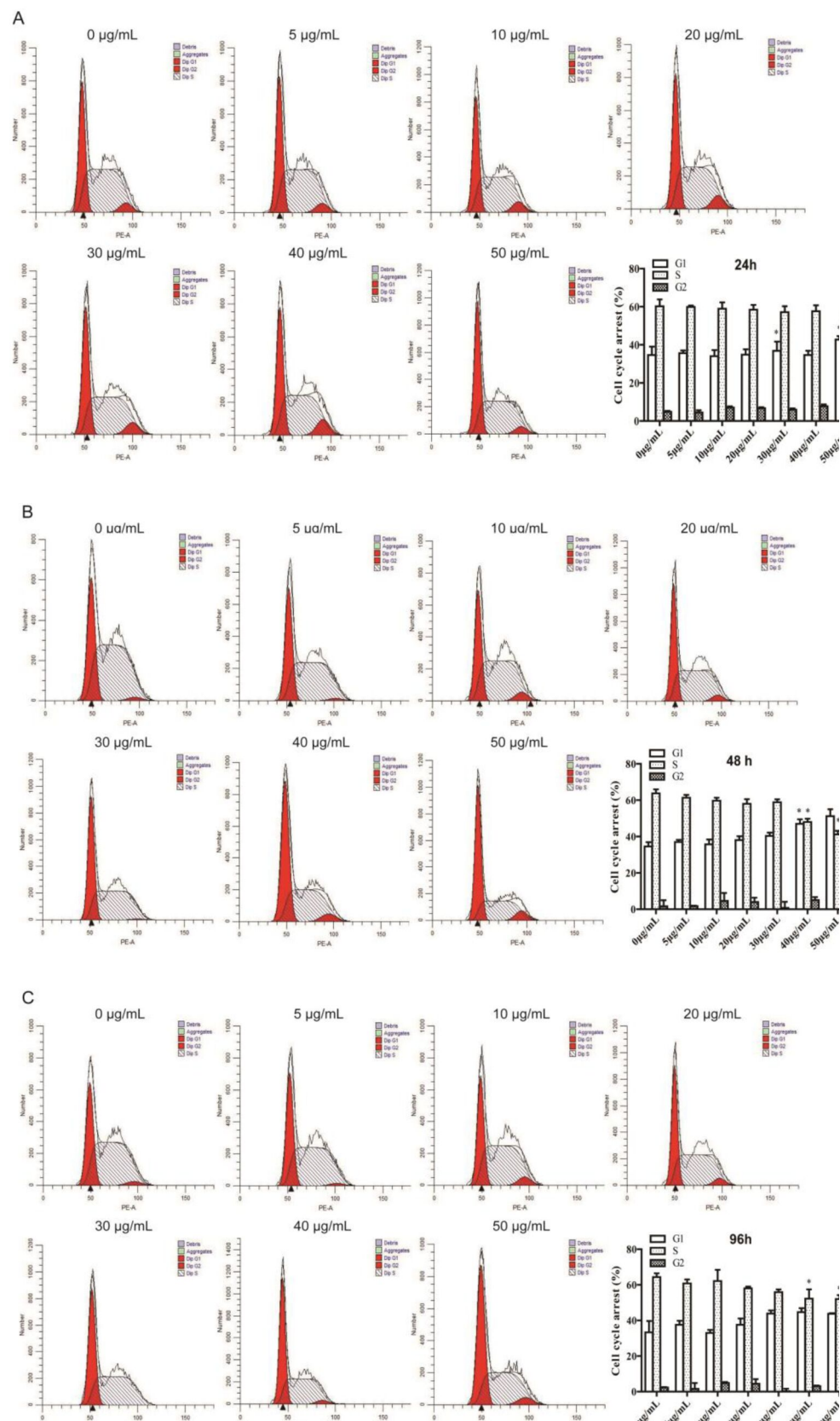

Figure S2

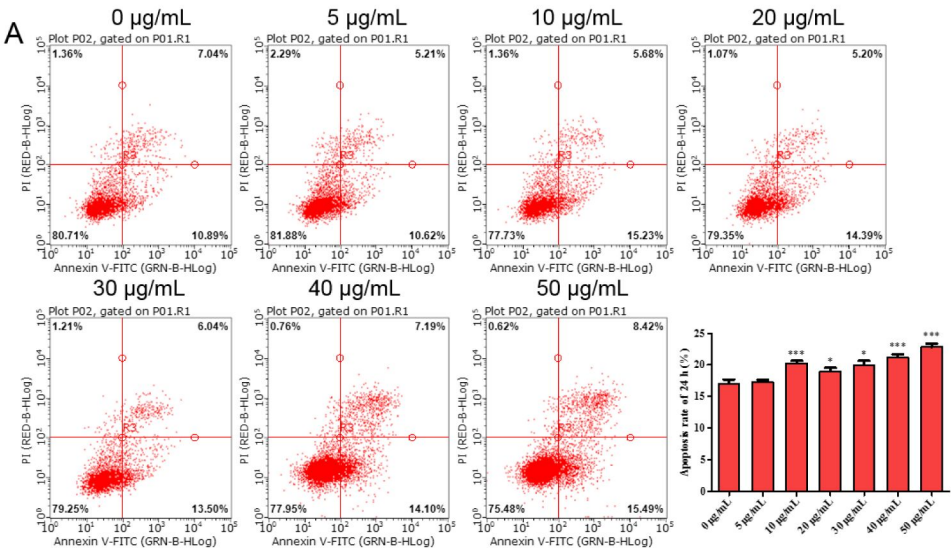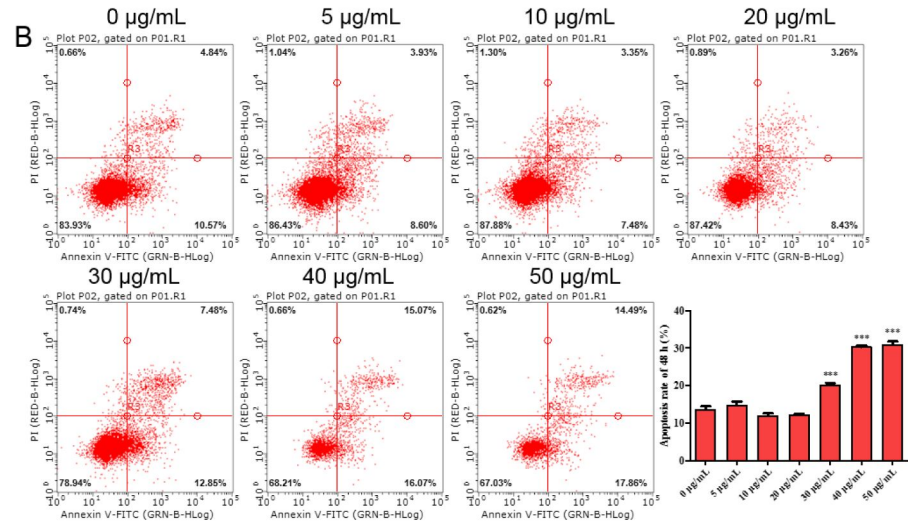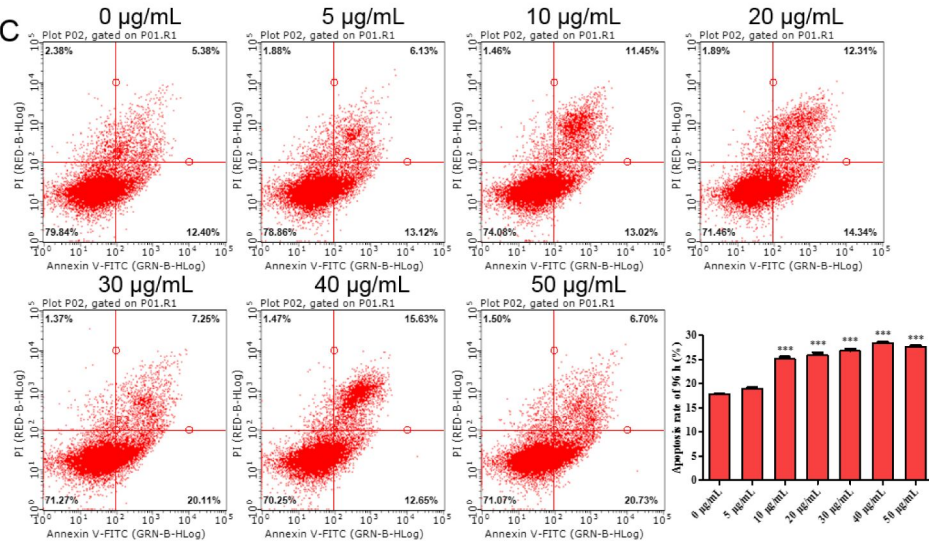

**Figure S2**

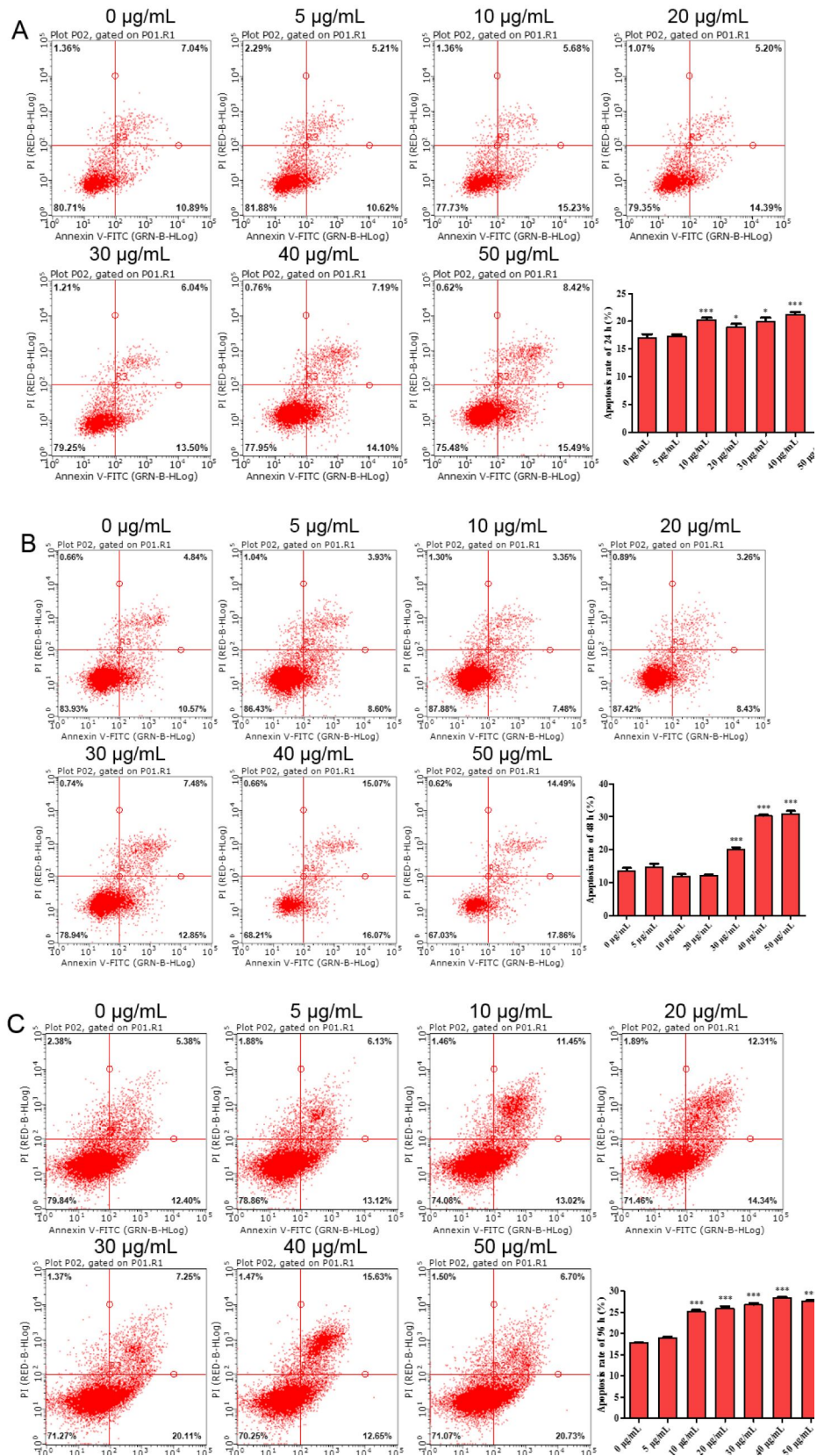

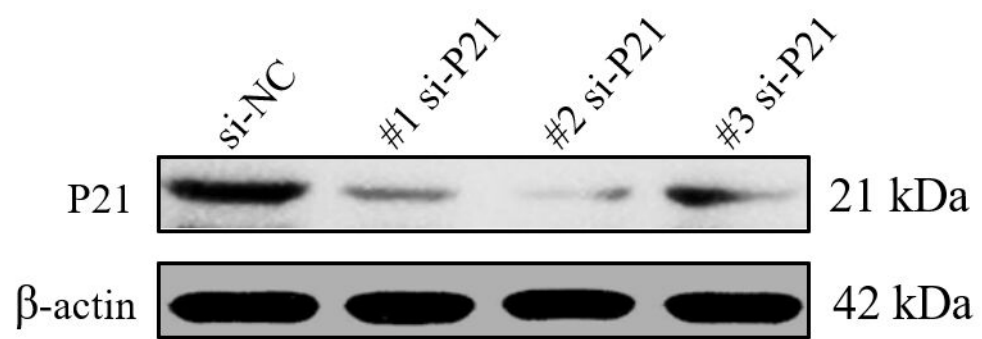

Supplement: Supplementary file 1 [file medi-101-e29760-s001.pdf]
